# Isolation and Functional Analysis of *ZmLTP3*, a Homologue to *Arabidopsis LTP3*

**DOI:** 10.3390/ijms14035025

**Published:** 2013-03-01

**Authors:** Hua-Wen Zou, Xiao-Hai Tian, Guo-Hui Ma, Zhi-Xin Li

**Affiliations:** 1 College of Agriculture, Yangtze University, Jingzhou 434023, China; E-Mails: xiaohait@sina.com (X.-H.T.); lizhixin09@163.com (Z.-X.L.); 2 China National Hybrid Rice Research and Development Center, Changsha 410125, China; E-Mail: maguohui@hhrrc.ac.cn

**Keywords:** *ZmLTP3*, transgenic *Arabidopsis*, salt resistance, morphological traits, physiological traits

## Abstract

Plant lipid transfer proteins (LTPs) are encoded by multigene families and play important roles in plant physiology. One full-length cDNA encoding an *Arabidopsis LTP3* homologue was isolated from maize by RT-PCR and named as *ZmLTP3*. RT-PCR analysis indicated that the *ZmLTP3* expression is induced by salicylic acid (SA), mannitol and salt. Furthermore, in different tissues the *ZmLTP3* displayed different expression patterns, indicating that *ZmLTP3* may play multiple roles in stress resistance. Over-expression of *ZmLTP3* in wild-type *Arabidopsis* resulted in the increased salt tolerance. Under salt stress condition, compared to wild-type (WT) plants, transgenic *Arabidopsis* grew better, had higher seedling fresh (FW), dry weight (DW), seed yields, proline content and lower MDA content and relative electric conductivity level. Our results suggest that maize *ZmLTP3* might encode a member of LTPs family and play roles in salt resistance.

## 1. Introduction

Plant lipid transfer proteins (LTPs) are a class of abundant, small (usually 6.5 to 10.5 kDa), ubiquitous and basic (pI = 8.5~12) proteins that are capable of transferring phospholipids and fatty acids between artificial membranes *in vitro*[1–3]. LTPs are mainly classified into two subfamilies, which are referred to as LTP1 and LTP2. LTP1 members contain 90 to 95 amino acid residues with molecular masses of approximately 9 kD, while LTP2 members contain approximately 70 amino acid residues with molecular masses of approximately 7 kD [4]. The structure of plant LTPs family is highly conserved. The LTPs proteins contain eight cysteine residues with four conserved disulfide bridges and two consensus pentapeptide motifs (Thr/Ser-X1-X2-Asp-Arg/Lys and Pro-Tyr-X-Ile-Ser) [5]. There are four α-helices in the three-dimensional structure of LTPs. These four α-helices form a hydrophobic cavity which runs through the whole protein molecule and can interact with fatty acids and phospholipid molecules *in vitro*[6].

LTPs are widely distributed in the plant kingdom. To date a large number of LTPs have been characterized from diverse species, such as *Arabidopsis*, cotton, wheat, rice, tobacco and so on [7–12]. Besides mediating phospholipid transfer, various biological functions of plant LTPs have been identified such as wax assembly, seed storage lipid mobilization, cuticle synthesis, somatic cell development, pollen tube adhesion and so on [4,13–16]. However, studies also show that LTPs play pivotal roles in plant defense mechanisms [17,18]. Due to their important roles in biotic stresses, LTPs are named as pathogenesis-related proteins and constitute the PR-14 family [19]. Compared to the roles in biotic stresses, the direct roles of LTPs in abiotic stresses are seldom reported.

In previous work, we have cloned a *Pti1*-like gene from maize named as *ZmPti1*[20]. *Arabidopsis* over-expressing *ZmPti1* gene displayed higher salt resistance [21]. In the follow-up study, microarray analysis was conducted to study the genes expression in transgenic *Arabidopsis.* As a result, the expression level of *LTP3* (At5g59320) was found to increase dramatically in transgenic line; *i.e.*, 17,792 fold in the transgenic line than in the wild type line. This indicates that LTP3 maybe a downstream component of Pto/Pti1 signal pathway and play roles in abiotic stresses.

In this study, we report the cloning of *ZmLTP3* (GenBank Accession No. JX435819), an *Arabidopsis LTP3* homologue from maize. The expression pattern of *ZmLTP3* in various maize tissues and also the seedlings treated by various stresses was studied. The functional analysis of *ZmLTP3* was conducted via transgenic *Arabisopsis*.

## 2. Results

### 2.1. Isolation and Sequence Analysis of *ZmLTP3*

**Figure 1:**
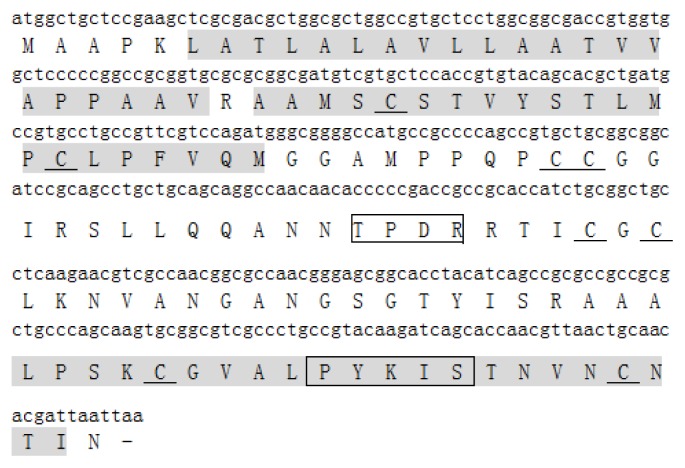
Sequence analysis of *ZmLTP3*. The predicted transmembrane regions are shown within gray boxes. Two sequences in frames indicate two consensus pentapeptides, and the underlined amino acids indicate the eight conserved cysteine residues.

The maize full-length cDNA clone ZM_BFc0160J05 was analyzed in ExPASy (http://au.expasy.org/tools/dna.html) and NCBI (http://blast.ncbi.nlm.nih.gov/) to confirm whether it contains a full-length *LTPs*-like cDNA [22,23]. The results showed that it is a full-length *LTPs*-like cDNA (data not shown). RT-PCR was then performed to clone this full-length cDNA sequence. Analysis based on the sequencing result of RT-PCR product indicated that the deduced protein, ZmLTP3, contains 123 amino acids with an estimated molecular mass of 12.5 kDa and an isoelectric point of 9.38. Using the PlantsP program, two predicted transmembrane region was found in ZmLTP3. Multiple sequence alignment of ZmLTP3 protein sequence with other related sequences showed that ZmLTP3 has the highest identities with a wheat type 1 non-specific lipid transfer protein precursor (GenBank Accession No. AJ852546.1) indicating it belongs to type 1 subfamily (data not shown). Additionally, eight conserved cysteine residues, two consensus pentapeptides: T/S-X-X-D-R/K and P-Y-X-I-S were also found in ZmLTP3 (Figure 1).

**Figure 2:**
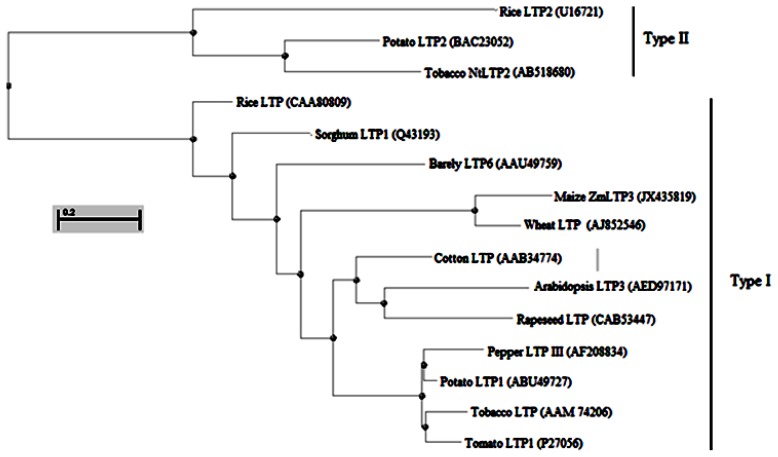
A phylogenetic tree of maize *ZmLTP3* with other plant LTP genes. This tree was constructed using the neighbor-joining method.

Phylogenetic analysis of the amino acid sequences of plant LTPs revealed that ZmLTP3 and a wheat type 1 non-specific lipid transfer protein precursor (GenBank Accession No. AJ852546.1) are grouped together with another type I family (Figure 2). These above analyses strongly suggest that the ZmLTP3 may encode a functional homologue of LTP.

### 2.2. Expression Analysis of *ZmLTP3*

**Figure 3:**
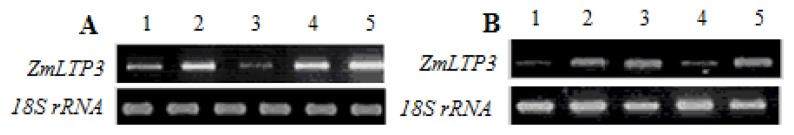
Expression of the *ZmLTP3* gene in various tissues and in response to various treatments. (**A**) Expression of *ZmLTP3* in various tissues. 1–5 shows different tissues: (1) roots, (2) coleoptiles, (3) leaves, (4) silks and (5) ovaries; (**B**) Expression of *ZmLTP3* under various treatments: (1) control, (2) mannitol, (3) salt, (4) 4 °C and (5) SA.

Expression pattern of *ZmLTP3* in different tissues and under various treatments were analyzed from maize. The results showed that the expression profiles of *ZmLTP3* in various tissues are significantly diverse. The expression levels of *ZmLTP3* are lowest in leaves, lower in roots, higher in coleoptiles and silks, highest in ovaries (Figure 3A). Figure 3B shows the expression pattern of *ZmLTP3* in response to various treatments. From the results we can see the *ZmLTP3* is induced by mannitol, salt and SA treatments, but not induced by cold treatment, indicating its involvements in both biotic and abiotic stresses signal pathway.

### 2.3. Salt Tolerance of Transgenic *Arabidopsis*

**Figure 4:**
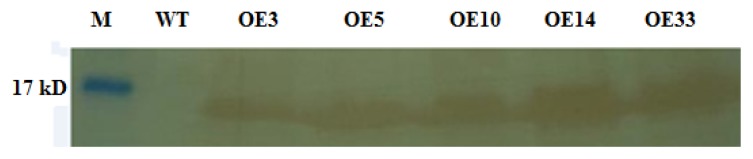
Western blot analysis of the *ZmLTP3* gene expression in wild type and homozygous transgenic lines. M, protein molecular weight marker; WT, wild type *Arabidopsis*; OE3, OE5, OE10, OE14 and OE33, transgenic lines.

The transformants were characterized by kanamycin and then PCR (data not shown). Only those PCR positive plants were allowed to produce homozygous transformants. The homozygous transformants were then used for Westen-blot analysis. As shown in Figure 4, all the PCR positive plants had a specific HA-reactive band with the molecular mass of about 13 kD. This not only showed that the PCR positive plants were transgenic plants but also showed that *ZmLTP3-dHA* fusion protein had been successfully expressed in transgenic transformants.

**Figure 5:**
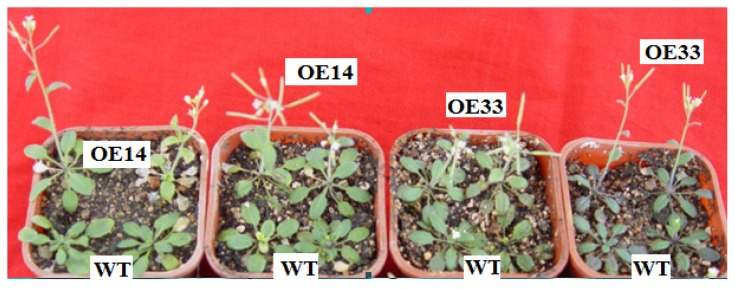
Effects of salt stress on growth of transgenic plants. WT, wild type *Arabidopsis*; OE14 and OE33, transgenic lines.

*ZmLTP3*-overexpressing *Arabidopsis* homozygous lines (OE14 and OE33), as well as the WT controls, were used for the examination of salt tolerance. After two weeks of 300 mM salt treatment, almost all the transgenic plants survived, while 71% WT plants survived. Furthermore, 68% OE14 plants and 65% OE33 plants could flower and set seeds, while the rate of WT plants was 34% (Figure 5). This result indicated that over-expression of *ZmLTP3* in *Arabidopsis* could increase salt tolerance.

### 2.4. Morphological and Physiological Analysis of Transgenic *Arabidopsis*

**Figure 6:**
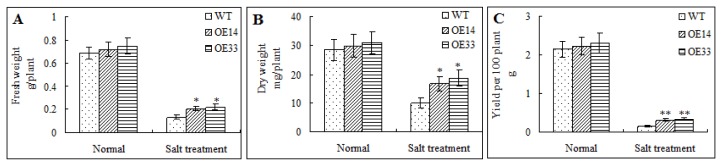
Morphological analysis of transgenic and WT plants under normal or salt treatment. (**A**) FW of plants treated with 0 mM NaCl or 300 mM NaCl; (**B**) DW of plants treated with 0 mM NaCl or 300 mM NaCl; (**C**) seeds weight of plants treated with 0 mM NaCl or 300 mM NaCl. Error bars indicate ± SE (*n* = 3). * and **, Significantly different from the WT at *p* < 0.05 and < 0.01, respectively, by Student’s *t* test.

Without salt stress, the wild-type and transgenic *Arabidopsis* plants displayed no differences in their FW, DW and seeds weight. Under salt stress, the FW, DW and seeds weight content in each plant were decreased. Interestingly, the FW, DW and seeds weight of two transgenic lines were significantly higher than that of WT plants (Figure 6). This result showed that over-expression of *ZmLTP3* enhanced both biomass and economic yields in transgenic *Arabidopsis* under salt treatment.

**Figure 7:**
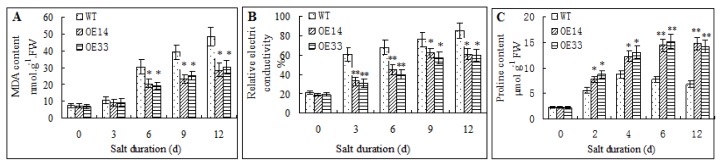
Physiological analysis of transgenic and WT plants under normal or salt treatment. (**A**) MDA content in plants treated with 0 mM NaCl or 300 mM NaCl; (**B**) Ion leakage ration of plants treated with 0 mM NaCl or 300 mM NaCl; (**C**) Proline content in plants treated with 0 mM NaCl or 300 mM NaCl. Error bars indicate ± SE (*n* = 3). * and **, Significantly different from the WT at *p* < 0.05 and < 0.01, respectively, by Student’s *t* test.

Lipid peroxidation and ion leakage are often resulted from salt stress [24]. In this study, MDA content and ion leakage ratio in transgenic and WT plants were analyzed. Under normal conditions (0 day), The *ZmLTP3*-overexpressing *Arabidopsis* and WT plants exhibited no significant differences in the MDA content and ion leakage ratio. When treated with salt stress, MDA content and ion leakage ratio increased in both transgenic and WT plants, but their improvements in WT plants were significantly higher than that in transgenic plants (Figure 7A,B). In contrast, the proline content in transgenic plants was significantly higher than that in WT plants under salt stress condition. After 4 days’ of salt treatment, in the transgenic *Arabidopsis* plants, the proline level was over two-fold higher than the WT control (Figure 7C). These results showed that over-expression of *ZmLTP3* could decrease MDA content and ion leakage, while increase proline content under salt treatment in *Arabidopsis*.

## 3. Discussion

In this report, a novel maize gene, *ZmLTP3* was PCR cloned and the deduced protein contains 123 amino acids with about MW 12.5 kDa and pI 9.38. The gene has 81% identities at the DNA level and 72% at protein level with a wheat type 1 non-specific lipid transfer protein precursor (GenBank Accession no. AJ852546.1). Sequence analysis showed that the deduced protein contains eight conserved cysteine residues and two consensus pentapeptides: T/S-X-X-D-R/K and P-Y-X-I-S, which are thought to be present in the 9-kDa type 1 subfamily and play a catalytic role or a role in lipid binding [5]. Expression pattern showed that the expression profiles of *ZmLTP3* differ in different tissues and can be up-regulated by not only SA, but also salt and mannitol, which indicated that *ZmLTP3* may not only be responsible for biotic stress but also, may be responsible for abiotic stress. Taken together, we speculate that *ZmLTP3* encodes a protein which belongs to type 1 subfamily and may play roles in both biotic and abiotic stresses.

RT-PCR analysis showed that the expression level of *ZmLTP3* is highest in ovaries among all the checked tissues. We are interested in whether it influences the developments of reproductive organs. The sizes of flowers and seeds, and the average number of seeds in siliques in both WT and transgenic plants were checked under normal condition. Unfortunately, no obvious differences were found (data not shown). Maybe it plays roles in other physiology processes, which needs to be further analyzed in the follow-up study. To investigate whether *ZmLTP3* plays a role in salt resistance as does *ZmPti1*, *ZmLTP3*-overexpressing transgenic *Arabidopsis* was characterized. Phenotypically, there were no obvious differences, including the sizes of plants, the flower time, and so on between WT and transgenic plants under normal condition. But, under salt conditions, the transgenic plants grew differently from the WT plants. Both transgenic lines exhibited earlier and more flowering properties than the WT plants under stress condition. The earlier flower time of transgenic plants maybe result from its interaction with key floral transcription repressors such as FLOWERING LOCUS T (FT) and/or other components of the autonomous flowering pathway. Because biomass and economic yields are useful traits for stress tolerance evaluation, in this study, the FW, DW and seeds weight of transgenic and WT plants were measured in both normal and salt stress conditions. Under normal condition, there were no obvious differences among the plants. When treated with salt, FW and DW of transgenic plants were significantly higher than that in WT plants, furthermore, the seeds weight of transgenic plants showed over two-fold increase in comparison with that of WT plants, suggesting that the over-expression of *ZmLTP3* in *Arabidopsis* led to an increased salt resistance. These results strongly demonstrate that the biological function of *ZmLTP3* is closely related to salt resistance in plants.

Some physiological traits related to salt-resistance were further measured to explore the mechanisms of the possible *ZmLTP3*-mediated salt resistance. MDA content and relative electric conductivity are important indicators for cell membrane injury caused by the oxidative stress under stresses [24]. In this study, *ZmLTP3*-overexpressing *Arabidopsis* contained a lower level of MDA content and ion leakage ratio under salt stress. In addition, it is well known that as an organic solute, proline accumulation can increase the osmotic pressure and, thus, improve the salt tolerance in plants [25]. In this study, the proline content in *ZmLTP3*-overexpressing *Arabidopsis* was higher than that in the WT plants under salt treatment, indicating its higher osmotic regulation capacity. Taken together, all these data showed that Zm*LTP3* might increase the salt resistance by maintaining the stability of membrane and minimizing dehydration in living cells.

## 4. Experimental Section

### 4.1. Maize Seedlings Growth Conditions and Treatments

Plant material, growth conditions and experimental treatments were performed as described by Zou *et al.*[20]. In brief, the germinating maize seeds were sown on a layer of hydrophilic cotton in trays and covered with two layers of wet hydrophilic pledget. When the primary roots were about 1.5–2 cm, the roots and coleoptiles of some seedlings were harvested for RNA extraction for RT-PCR analysis, Other left seedlings were transferred to a chamber with 70% relative air humidity, 30 °C/28 °C day/night temperature, a day/night cycle of 16 h/8 h and a 300 μmol m^−2^ s^−1^ photon flux density. When the ligules of the second leaf were visible the seedlings were subjected to the following treatments. SA treatment was conducted by spraying with 2 mM SA; mannitol and NaCl treatments were performed by transferring the seedlings onto the trays watered with 600 mM mannitol and 150 mM NaCl solution; Low-temperature treatment was conducted by transferring the seedlings onto a tray with water pre-equilibrated at 4 °C in a growth chamber for 24 h prior to the treatment. The seedlings were treated at 4 °C under illumination and air humidity conditions as above. 24 h after treatments, the leaves were harvested from all the above treated seedlings for RT-PCR analysis. Mature leaves and ovaries were sampled from field-grown maize. Ovaries were excised from the central part of the ears before fertilization, and mature leaves were also sampled at the same time.

### 4.2. Cloning of *ZmLTP3* cDNA

The sequence of *LTP3* (At5g59320) was used as the query probe to search homologues in maize in NCBI database. A highly homologous maize full-length cDNA clone ZM_BFc0160J05 was obtained for analysis of motifs/domain in PlantsP database. According to the sequence, two primers, 5′-ACGCGTCTATCTCAGCTTGCTG-3′ (forward primer) and 5′-GGCAACATGCATCCACGATACCAC-3′ (reverse primer) were used to amplify cDNA of *ZmLTP3* via RT-PCR from 150 mM NaCl treated maize seedling leaves. RNA was isolated using TRIzol Reagent (Invitrogen, Carlsbad, CA, USA). The RT-PCR procedure was performed according to Zou *et al.*[20].

### 4.3. RT-PCR Analysis of *ZmLTP3*

RT-PCR was used to semi-quantitatively determine the expression profile of the *ZmLTP3* gene. 5 μg RNA from different sample was reverse-transcribed to produce cDNA. The specific primers (5′-ATGGCTGCTCCGAAGCTC-3′, sense primer; 5′-TGCAGTTAACGTTGGTGC-3′, antisense primer) were used for RT-PCR analysis. The expected length of the amplified fragment is 358 bp. The total volume of PCR reaction was 25 μL, containing 1 μL of the first strand cDNA, 0.4 μM of each primer, 1×PCR reaction buffer, 0.2 mM of dNTP and 1 U of Taq DNA polymerase. The reaction was denatured at 95 °C for 3 min, and then subjected to 25 cycles of 95 °C 30 s, 56 °C 30 s and 72 °C 1 min, plus a final extension at 72 °C for 5 min. The PCR products were separated on 1.5% agarose gel and quantified scanned using the higher performance ultraviolet transilluminator (GDS-8000, Gel Documentation System, UVP, Upland, CA, USA). Maize *18S rRNA*, amplified with primers 5′-CCATAAACGATGCCGA-3′ (sense primer) and 5′-CACCACCCATAGAATCAAGA-3′ (antisense primer), was used as the internal standard in the experiment. The experiments were repeated three times with the similar results and one of them was presented.

### 4.4. Plasimid Construction, *Arabidopsis* Transformation and Transformants Characterization

The cDNA sequence of *ZmLTP3* in pGEM-T vector was amplified by PCR with adding BamH I and Stu I enzyme sites into 5′ and 3′ primers respectively. Then the PCR product was digested with *Bam*H I/Stu I. The expression vector p426GAL1 containing a fragment *ZmPti1-dHA* was digested with *Bam*H I and Stu I to release the *ZmPti1*. The digested products were ligated with T4 DNA ligase to produce recombinant vector p426GAL1 containing *ZmLTP3-dHA*. Then the recombinant vector p426GAL1 was digested with *Bam*H I and Pst I to release *ZmLTP3-dHA.* The expression vector pGreen0029 containing a fragment *35S-C4DDPK-CBF3-NOS* was also digested with *Bam*H I and *Pst* I to release the *CBF3*. The digested products were ligated with T4 DNA ligase. Then the recombinant vector pGreen0029 containing the fragment *35S-C4DDPK-ZmLTP3-dHA-NOS* was successfully constructed after sequencing confirmation. The *Arabidopsis* transformation and the following characterization of transformants were performed as described previously [21], except that the primers specific to *ZmLTP3* was used here to amplify by PCR.

### 4.5. Arabidopsis Growth Conditions and Stress Treatments

*Arabidopsis* growth conditions and salt treatment to pot-grown *Arabidopsis* were performed as described by Zou *et al.*[21].

### 4.6. Morphological and Physiological Traits Measurements

After two weeks of salt treatment, the plants were photographed; some plants were harvested for seedling fresh and dry weight, other plants restored to normal growth condition. The fresh weight of whole plants was measured immediately after harvesting. Dry weight was measured after 48 h at 80 °C. When all the other plants restored to normal growth condition maturated the seeds of WT and transgenic plants were harvested and weighed. Three replicates (100 plants per replicate) per line were used in morphological assays. Physiological assays were conducted at 0, 3, 6, 9, 12 days, respectively, after salt treatments. Proline content was determined spectrophotometrically at 520 nm following the ninhydrin method described by Bates *et al.*[26]. MDA was determined by a color reaction with thiobarbituric acid [27]. For relative electric conductivity parameter measurement, 0.1 g same positional leaves were removed from different plants, rinsed briefly with deionized water and immediately placed into a tube with 10 ml of deionized water. Conductivity (*I*_1_) was measured using an electro conductivity meter (model 1054, VWR Scientific, Radnor, PA, USA) after the tubes were placed at 22 °C overnight. Then, the samples were heated at 100 °C for 30 min and conductivity (*I*_2_) was measured again. Ion leakage ratio was expressed as (*I*_1_/*I*_2_) ×100%. For all the above measurements, three replicates per line were used. Statistical differences were determined using Student’s two-tailed *t* test.

## 5. Conclusions

In conclusion, we have successfully cloned *ZmLTP3* cDNA from maize which is a homologue to *Arabidopsis*. Sequence analysis showed that it belongs to the type 1 subfamily of LTPs. Different levels of *ZmLTP3* expression was found in different tissues; moreover, *ZmLTP3* were induced by both biotic and abiotic stresses. Over-expression of *ZmLTP3* in *Arabidopsis* resulted in the improved salt resistance.
